# Transcriptome analysis reveals the genetic basis underlying the biosynthesis of volatile oil, gingerols, and diarylheptanoids in ginger (*Zingiber officinale* Rosc.)

**DOI:** 10.1186/s40529-017-0195-5

**Published:** 2017-10-23

**Authors:** Yusong Jiang, Qinhong Liao, Yong Zou, Yiqing Liu, Jianbin Lan

**Affiliations:** Research Institute for Special Plants, Chongqing University of Arts and Sciences, Chongqing, 402160 China

**Keywords:** Ginger, Transcriptome sequencing, Rhizome, Fibrous root, Bioactive compounds

## Abstract

**Background:**

Ginger (*Zingiber officinale* Rosc.) is a popular flavoring that widely used in Asian, and the volatile oil in ginger rhizomes adds a special fragrance and taste to foods. The bioactive compounds in ginger, such as gingerols, diarylheptanoids, and flavonoids, are of significant value to human health because of their anticancer, anti-oxidant, and anti-inflammatory properties. However, as a non-model plant, knowledge about the genome sequences of ginger is extremely limited, and this limits molecular studies on this plant. In this study, de novo transcriptome sequencing was performed to investigate the expression of genes associated with the biosynthesis of major bioactive compounds in matured ginger rhizome (MG), young ginger rhizome (YG), and fibrous roots of ginger (FR).

**Results:**

A total of 361,876 unigenes were generated by de novo assembly. The expression of genes involved in the pathways responsible for the biosynthesis of major bioactive compounds differed between tissues (MG, YG, and FR). Two pathways that give rise to volatile oil, gingerols, and diarylheptanoids, the “terpenoid backbone biosynthesis” and “stilbenoid, diarylheptanoid and gingerol biosynthesis” pathways, were significantly enriched (adjusted *P* value < 0.05) for differentially expressed genes (DEGs) (FDR < 0.005) both between the FR and YG libraries, and the FR and MG libraries. Most of the unigenes mapped in these two pathways, including curcumin synthase, phenylpropanoylacetyl-CoA synthase, trans-cinnamate 4-monooxygenase, and 4-hydroxy-3-methylbut-2-en-1-yl diphosphate synthase, were expressed to a significantly higher level (log_2_ (fold-change) ≥ 1) in FR than in YG or MG.

**Conclusion:**

This study provides the first insight into the biosynthesis of bioactive compounds in ginger at a molecular level and provides valuable genome resources for future molecular studies on ginger. Moreover, our results establish that bioactive compounds in ginger may predominantly synthesized in the root and then transported to rhizomes, where they accumulate.

**Electronic supplementary material:**

The online version of this article (doi:10.1186/s40529-017-0195-5) contains supplementary material, which is available to authorized users.

## Background

Ginger (*Zingiber officinale* Rosc.), which has a cultivar history more than 2000 years in China, is now a popular condiment and medicine all over the world. The distinct flavor of the ginger rhizome makes it popular as a food additive. In traditional Chinese medicine (TCM), ginger is used to relieve nausea and vomiting, eliminate phlegm and relieve cough, improve the internal environment by clearing heat and dampness, and strengthen the immune system (Chang et al. 2011; Li 1985; Qiang et al. 2009; Therkleson 2010; Wang and Wang 2005). In recent years, clinical studies have shown that ginger is an effective medicine for treating degenerative disorders, indigestion, cardiovascular disorders, vomiting, diabetes mellitus, and cancer (Jiang et al. 2006; Mashhadi et al. 2013; Nicoll and Henein 2009; Shukla and Singh 2007; Wei et al. 2005; Weng et al. 2010). Moreover, it is generally accepted that ginger has anti-inflammatory properties and functions as an anti-oxidant by scavenging reactive oxygen species (ROS) in cells, which cause human diseases and aging (Dugasani et al. 2010; Mishra et al. 2007; Sekiwa et al. 2000; Torkzadeh-Mahani et al. 2014; Zheng and Wang 2001). Understanding the molecular basis of the biosynthesis of pharmacologically active compounds in ginger is of great practical value for molecular breeding and medicinal use of this plant.

Chemical component analysis has been used to identify hundreds of compounds in ginger. These compounds can generally be classified into three categories: volatile oils, gingerols, and diarylheptanoids (Afzal et al. 2001; Ding and Ding 1988; Jiang et al. 2006). These three categories of compounds contribute to the pungency, biological actions and pharmacological properties. The volatile oil contains sesquiterpenoids and monoterpenoids. Gingerols are a homologous series of phenols that include shogaols, paradols, and gingerone. (Semwal et al. 2015). Diarylheptanoids is a class of derivatives that have a 1,7-diarylheptane skeleton, including curcuminoids, which are compounds found in turmeric (Jiang et al. 2007). In addition to these compounds, ginger contains a large amount of flavonoids, which are natural antioxidants that can lower the risk of cancer, high blood pressure, and heart disease (Ghasemzadeh et al. 2010a, b; Knekt et al. 2002).

Previous studies on ginger have mainly focused on chemical and pharmacological analyses or the products obtained by processing ginger. Little is known about the genome sequences and molecular bases of the biological processes in ginger. In this study, we explored the gene expression profiles of rhizomes and roots, and examined the molecular bases underlying biosynthesis of the major bioactive compounds in ginger. To accomplish this, we *de novo* sequenced the transcriptomes of matured ginger rhizome, young ginger rhizome, and the fibrous roots of the ginger cultivar, Yujiang1. The transcripts obtained in the present study will add to the available genome resources for *Zingiberaceae* plants and provide molecular information for use in molecular breeding and biological study of ginger.

## Methods

### Ginger material

The ginger variety, Yujiang1, was grown under normal field conditions in Chongqing, China (29°14′ N, 105°52′ E). The sampling were carried out at the vigorous growth and developmental stage, i.e. 90 days after planting. The healthy rhizomes were collected, surface cleaned, and drained. The matured and young ginger from the same plant were than crosscut into slices and frozen in liquid nitrogen, respectively. Matured ginger rhizomes (MG) are ginger rhizomes that harvested at last year and used as planted propagule, and the young ginger rhizomes (YG) are newly formed and immature ginger rhizomes in present year. The fibrous roots in young ginger were immediately frozen in liquid nitrogen after harvest. All samples were stored at −80 °C.

### RNA isolation, cDNA library construction and sequencing

Total RNA of matured ginger rhizome (MG, the planted propagule), young ginger rhizome (YG) and fibrous roots (FR) was isolated using RNAprep Pure Plant kits (Bio TeKe, China). The RNA concentration and quality were determined by a Qubit 3.0 (Thermo Scientific, USA) and an Agilent 2100 Bioanalyzer (Agilent Technologies, USA), respectively. For each tissue (MG, YG, and FR), total RNA from five individual plants was pooled for library construction. cDNA libraries were prepared using a NEBNext Ultra Directional RNA Library Prep kit (cat#E7420, NEB, UK) for strand-specific RNA-seq. Briefly, the mRNA was first fragmented and fragments of 300–500 bp were enriched with magnetic beads. The first cDNA strands were then biosynthesized from the fragments. The second cDNA strands were subsequently synthesized by adding dUTP as a marker. Finally, the double strand cDNA was subjected to digestion with uracil-DNA glycosylase (UDG) before PCR reaction and transcriptome sequencing. In this way, only the first strands of cDNA were retained and sequenced. Libraries were sequenced on an Illumina Hiseq 2500 platform using a paired-end run (2 × 150 bp).

### Transcriptome assembly and functional annotation

The raw reads generated for each library were quality-filtered by removing adapter sequences and reads with quality scores below Q20. The clean reads of the three libraries were de novo assembled using the Trinity Program (V 2.2.0) with default parameters (Grabherr et al. 2011). The transcriptomes were assembled using the *de Bruijn* strategy, and the longest transcript in each *de Bruijn* graph was defined as a unigene and used as reference sequence for assembly and coding sequence (CDS) prediction. The CDS and protein sequences of unigenes were predicted by TransDecoder (http://transdecoder.github.io/). For functional annotation, BlastX was used with an E-value threshold of 1 × 10^−5^ to align the unigenes to the following databases: the cluster of orthologous groups of proteins (COG) (https://www.ncbi.nlm.nih.gov/COG/), NCBI non-redundant proteins (NR) (https://www.ncbi.nlm.nih.gov/refseq/about/nonredundantproteins/), Swiss-Prot (http://www.uniprot.org/), gene ontology (GO) (http://www.geneontology.org/) and kyoto encyclopedia of genes and genomes (KEGG) (http://www.genome.jp/kegg/pathway.htkl) databases.

### Gene expression measurement, GO and KEGG enrichment analysis

The expression levels of unigenes were calculated and normalized using the fragments per kilobase of exon per million fragments mapped (FPKM) method (Li and Dewey 2011). Significant differentially expressed genes (DEGs) between libraries were restricted according to Benjamini and Yekutieli (2001), with a false discovery rate (FDR) threshold < 0.005 and absolute value of log_2_(fold-change) ≥ 1. Significantly enriched GO terms in DEGs were defined as those with corrected *P* values < 0.05 when compared to the genome background, as described by Benjamini and Yekutieli (2001). KEGG Pathways with significant enrichment for DEGs were defined as those with Q values < 0.05.

### Validation of differential expression genes by qRT-PCR

Quantitative RT-PCR (qRT-PCR) was conducted to validate the expression levels of DEGs. cDNA of MG, YG and FR were generated using 1 μg of total RNA treated with an RNase-free DNase I set, followed by reverse transcription with a ReverAid First Strand cDNA Synthesis kit (Thermo Scientific, USA). Quantitative RT-PCR analysis was performed using a LightCycler 96 (Roch, USA) with SYBR Green master mix (BioRed, USA). Genes and primers are listed in Additional file 3: Table S1. Significantly different expression was determined by Tukey’s multi-comparison test (α = 0.05).

## Results and discussion

### Transcriptome data and assembly

A total of 18.05 G, 19.22 G, and 16.86 G clean data were generated for MG, YG, and FR, respectively (Table 1). The GC contents ranged from 49.51 to 51.19%, and the Q20 percentage ranged from 97.69 to 97.88% in clean reads. De novo assembly of the pooled cleaned reads was performed using Trinity based on chosen criteria. The assembled sequences were then used as reference for further analysis. The total mapped reads for MG, YG, and FR were 77.44, 75.15, and 73.83%, respectively. A total of 250,969, 240,132, and 225,366 unigenes were identified for the MG, YG, and FR libraries, respectively (Table 1). Finally, the *de novo* assembly generated a total of 361,876 unigenes, with an average length of 515.89 bp, and N50 length of 589 bp. The minimum length and maximum length was 201 bp and the maximum length was 21,690 bp, respectively (Table 2). Length distribution showed that 105,683 (29.2%) unigenes were longer than 500 bp (Additional file 1: Figure S1).

**Table 1:** Throughput and quality of strand specific RNA-seq of the three libraries

Library ID	Clean reads	Clean base (G)	Clean GC%	Clean Q20%	Total mapped reads (%)	Unigenes
MG	120,892,308	18.05	50.41	97.84	93,623,020 (77.44)	250,969
YG	128,809,296	19.22	49.51	97.88	96,789,876 (75.14)	240,132
FR	112,821,264	16.86	51.19	97.69	83,298,040 (73.83)	225,366

**Table 2:** Overview of the sequencing and assembly

Items	Number
Total unigenes	361,876
Average unigene length (bp)	515.89
Min unigene length (bp)	201
Max unigene length (bp)	21,690
N50 length (bp)	589
Total bases (bp)	186,687,182

Ginger plays important roles in the food processing and pharmaceutical industries. Many studies have focused on the chemical components and pharmacological effects of ginger (Hoferl et al. 2015; Li et al. 2016; Wang et al. 2015). However, the genome sequence of ginger is still unavailable. Only a limited number of expressed sequence tags (ESTs) for *Zingiber officinale* are available, with only 38,169 ESTs that can be retrieved from NCBI GenBank. The lack of genome information greatly limits molecular biology studies of ginger. The development of next-generation sequencing (NGS) technologies and bioinformatics has greatly facilitated studies on molecular biological changes and regulation at the genome-wide level, especially for non-model plants (Chai et al. 2009; Ward et al. 2012). The N50 value obtained for unigenes in the present study (589 bp) was lower than those reported by Prasath et al. (2014) (943 bp), and Gaur et al. (2016) (1251 bp). N50 length has been used as a criterion of transcriptome assembly quality; however, this value is greatly affected by the genome structure, assembly software, and assembly parameters (Li and Dewey 2011; Postnikova et al. 2013). Therefore, differences between studies in N50 values may be artifacts of the methods used for assembly. Thus, N50 values are not necessarily correlated with transcriptome accuracy. Our study generated 186.69 Mb of transcriptome sequences and the dataset will provide valuable information for future molecular studies of ginger and other Zingiberaceae plants. The raw data from this study are available in the NCBI database (SRA accession number SRP101401).

### Functional annotation and classification of unigenes

**Fig. 1:**
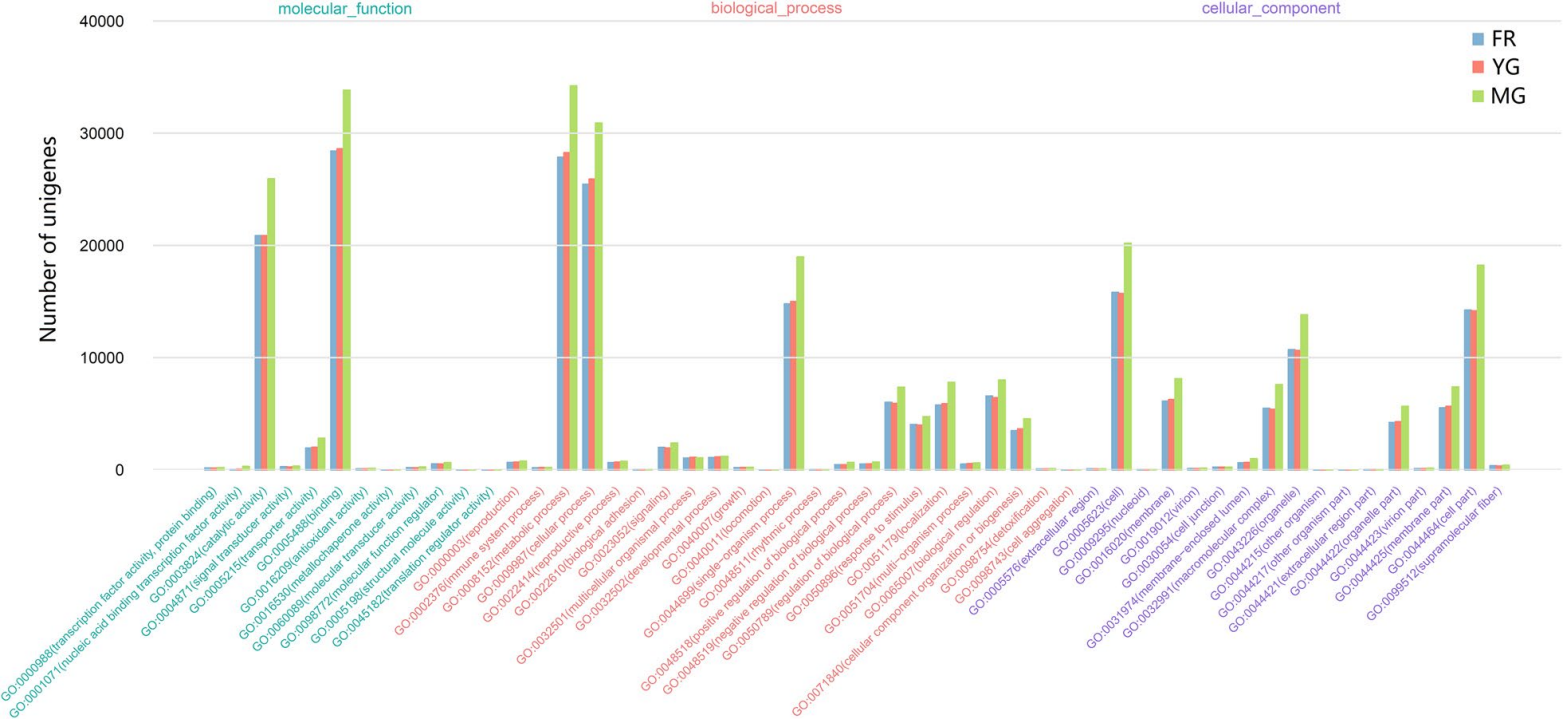
Histogram of gene ontology (GO) annotations for unigenes in ginger matured rhizome, young rhizome, and fibrous root libraries. Level two GO terms belonging to three categories, molecular function, biological process, and cellular component are along the x-axis and the number of unigenes assigned to each term is represented along the y-axis. Bars with different colors indicate different libraries

GO analysis was used to annotate 98,029 (39.06%), 91,092 (37.93%), and 81,695 (36.25%) of the unigenes in the MG, YG, and FR libraries, respectively. The distributions of the GO terms for the unigenes in each library were similar. The majority of the unigenes were contained in metabolic process (GO:0008152), binding (GO:0005488), cellular process (GO:0009987), catalytic activity (GO:0003824), cell (GO:0005623), cell part (GO:0044464), and single-organism process (GO:0044699) terms (Fig. 1). This suggests that there was no bias in library construction. A large number of the unigenes were classified into the metabolic process term, suggesting high metabolic activities in ginger rhizome and root. This may reflect the biosynthesis and accumulation of bioactive secondary metabolites in ginger.

Of the 361,876 unigenes identified, 184,768 (51.09%) were annotated in at least in one database (Additional file 2: Figure S2A): 99,726 (27.43%) were annotated with COG functions, 79,705 (22.02%) were annotated with KEGG enzymes, 177,366 (49.01%) were mapped to the NR database, and 122,191 (33.77%) were mapped to the Swiss-prot database. The alignment of unigenes against all proteins in the NR database showed that species with the greatest number of matches was *Musa acuminata* (28.38%), followed by *Gossypium* species (Gossypium hirsutum 9.64%, Gossypium raimondii 7.32%, Gossypium arboreum 2.32%; Additional file 2: Figure S2B). The unigenes identified in this study were not associated with any other individual species contained in the databases we examined. This is because of there was no genome data of Zingiberaceae species published to date (by searching the genome assembly information in the NCBI public database). This result indicates that the genome of ginger differs greatly from previously sequenced plant species. The big differences between the genomes of ginger and other species emphasizes the importance of genome and transcriptome sequencing of ginger or other Zingiberaceae plants in order to facilitate molecular studies of the whole genus.

### GO and KEGG enrichment of differentially expressed genes (DEGs)

Comparison of unigene expression levels across the three libraries showed that the number of unigenes with medium (3.75 < FPKM < 15) and high (FPKM > 15) expression levels in matured ginger (MG) (29,715, 8.22%) was lower than those in young ginger (YG) (36,789, 10.17%) and fibrous roots (FR) (38,466, 10.63%; Additional file 4: Table S2). The differences in expression levels between these tissues may therefore indicate that metabolic activity is more vigorous in newly generated tissues.

**Fig. 2:**
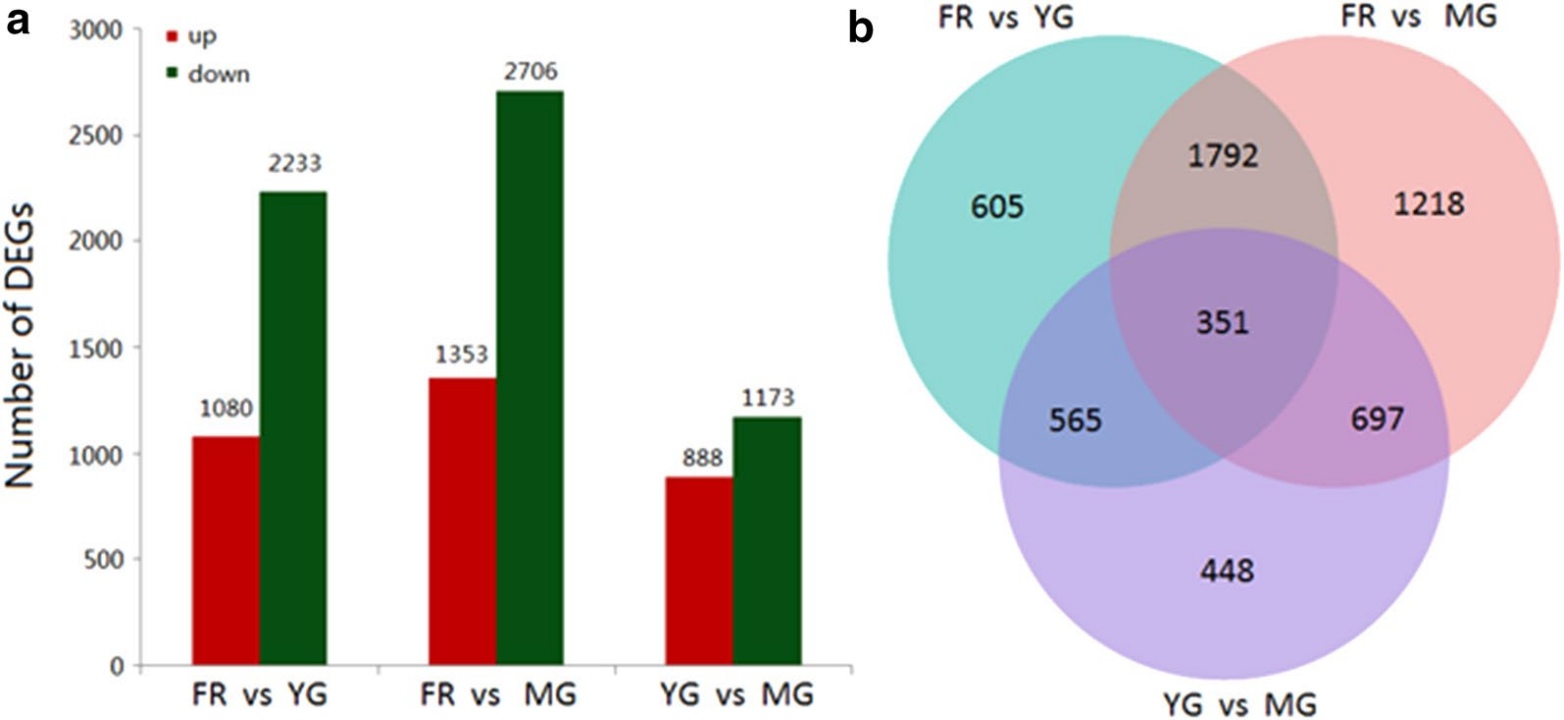
Pairwise comparisons of unigene expression in ginger matured rhizome, young rhizome, and fibrous root libraries (**a**) and a Venn diagram of the differentially expressed genes (DEGs) among the three libraries (**b**)

To identify DEGs, pairwise comparisons of unigene expression levels were performed between the FR and YG, FR and MG, and YG and MG libraries, (FDR < 0.005, log_2_ (fold change) ≥ 1). In comparison between FR and YG libraries, 1080 up-regulated and 2233 down-regulated DEGs were identified. In FR and MG, 1353 up-regulated and 2706 down-regulated DEGs were identified, and in YG and MG, 888 up-regulated and 1173 down-regulated DEGs were identified (Fig. 2a). There were many more DEGs between gingers rhizome tissues and fibrous root tissue (MG versus FR and YG versus FR) than there were between tissues from the different parts of ginger rhizomes (MG versus YG). This suggests a large difference in the expression of genes between ginger tissues. Comparison across the three libraries identified 351 DEGs (Fig. 2b).

**Fig. 3:**
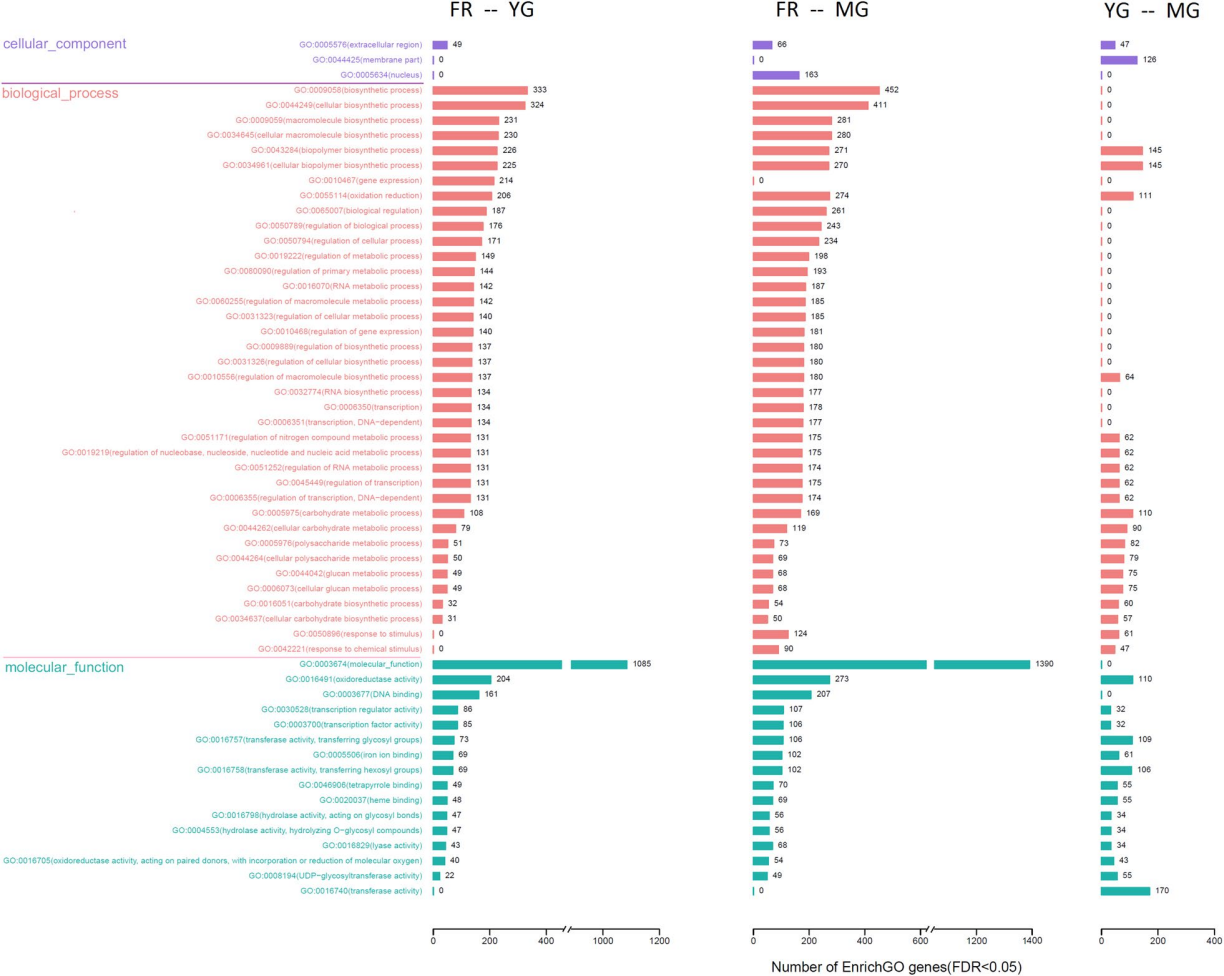
Gene ontology (GO) enrichment of differentially expressed genes (DEGs) between ginger matured rhizome, young rhizome, and fibrous root libraries. P values are represented along the x-axis and level two GO terms belonging to three categories, molecular function, biological process, and cellular component are represented along the y-axis. Numbers indicate the number of unigenes assigned to each term

GO classification of DEGs between the FR and YG, FR and MG, and YG and MG libraries resulted in assigning them to 52, 54, and 35 level two GO terms, respectively. The most enriched GO terms for DEGs between MG and FR were the same as those for DEGs between YG and FR. However, these terms were notably different from the most enriched GO terms for DEGs between MG and YG (Fig. 3). The “molecular function” (GO: 0009058) term in the molecular function category and many GO terms in the biological process category, which is mainly related to biosynthetic processes and metabolic processes, contained the largest numbers of DEGs between MG and FR and between YG and FR. However, no or few DEGs between MG and YG were assigned to these GO terms (Fig. 3).

KEGG classification identified a total of 49, 32, and 25 significantly enriched pathways (Q value < 0.05) for DEGs between the FR and MG, FR and YG, and MG and YG libraries, respectively (Additional file 5: Table S3). As with the GO term distributions, the majority of significantly enriched KEGG pathways for DEGs between FR and YG were similar to those between FR and MG libraries. Two pathways, the “terpenoid backbone biosynthesis” (ko00900) and “stilbenoid and diarylheptanoid and gingerol biosynthesis” (ko00945) pathways, were significantly enriched for DEGs between FR and MG, and FR and YG. These pathways give rise to the biosynthesis of volatile oil, gingerols, and diarylheptanoids in ginger (http://www.kegg.jp/kegg/pathway.html). Further KEGG enrichment for the 351 DEGs identified across the three libraries resulted in 16 significantly enriched pathways (P value < 0.05; Table 3). Among these pathways, the “phenylalanine metabolism” pathway (ko00360) and the “phenylpropanoid biosynthesis” pathway (ko00940) produces substrates for the “phenylpropanoid biosynthesis” pathway (ko00940), which generates substrates for the biosynthesis of pharmacologically active metabolites in ginger rhizomes (Ramirez-Ahumada et al. 2006).

**Table 3:** The significantly enriched KEGG pathways for DEGs across the three cDNA libraries

Pathway ID	Pathway	P value	Adjusted P value	DEG number (70)	All unigenes with pathway annotation (52,831)
ko00960	Tropane, piperidine and pyridine alkaloid biosynthesis	2.22497E−07	1.80223E−05	7 (10%)	316 (0.6%)
ko00360	Phenylalanine metabolism	0.000440777	0.017851483	6 (8.57%)	736 (1.39%)
ko04075	Plant hormone signal transduction	0.000867937	0.019222353	9 (12.86%)	1893 (3.58%)
ko00270	Cysteine and methionine metabolism	0.000949252	0.019222353	6 (8.57%)	853 (1.61%)
ko00940	Phenylpropanoid biosynthesis	0.001677001	0.027167422	6 (8.57%)	954 (1.81%)
ko00062	Fatty acid elongation in mitochondria	0.003175276	0.042866226	3 (4.29%)	220 (0.42%)
ko00130	Ubiquinone and other terpenoid-quinone biosynthesis	0.004387597	0.050770761	3 (4.29%)	247 (0.47%)
ko04626	Plant-pathogen interaction	0.007368338	0.07460442	8 (11.43%)	2145 (4.06%)
ko00592	alpha-Linolenic acid metabolism	0.016010692	0.144096225	3 (4.29%)	398 (0.75%)
ko04740	Olfactory transduction	0.026994683	0.197111092	2 (2.86%)	192 (0.36%)
ko00910	Nitrogen metabolism	0.028187756	0.197111092	3 (4.29%)	495 (0.93%)
ko04910	Insulin signaling pathway	0.030128861	0.197111092	5 (7.14%)	1312 (2.48%)
ko04744	Phototransduction	0.032099363	0.197111092	2 (2.86%)	211 (0.4%)
ko04971	Gastric acid secretion	0.034068584	0.197111092	2 (2.86%)	218 (0.41%)
ko04970	Salivary secretion	0.037551065	0.20277575	2 (2.86%)	230 (0.44%)
ko04745	Phototransduction—fly	0.048435888	0.245206684	2 (2.86%)	265 (0.5%)

The study by Zhan et al. (2009) showed that the major components of volatile oil, gingerols, and diarylheptanoids in FG and YG almost the same, while the contents of some of the components such as α-zingiberene, α-curcumene, and 6-gingerol were significant different. Our study provides the molecular evidence for the differences on the contents of bioactive compounds between ginger rhizomes with different mature degree. However, the content and biosynthesis of bioactive compounds in ginger fibrous root had not documented yet. In this study, the DEGs between FR vs. YG, and FR vs. MG libraries in the “terpenoid backbone biosynthesis” and “stilbenoid and diarylheptanoid and gingerol biosynthesis” pathways brings new insight into the material biosynthesis and metabolism between different ginger parts.

### Candidate genes involved in the biosynthesis of bioactive compounds in ginger

Volatile oil, gingerols and diarylheptanoids are three major categories of bioactive chemical compounds in ginger (Afzal et al. 2001; Kuo et al. 2005). In this study, two KEGG pathways that are related to the biosynthesis of these compounds, “terpenoid backbone biosynthesis” (ko00900) and “stilbenoid and diarylheptanoid and gingerol biosynthesis” (ko00945), were significantly enriched. Based on the NR annotation, a total of 33 unigenes were mapped to the “terpenoid backbone biosynthesis”, including 11 unigenes for 4-hydroxy-3-methylbut-2-en-1-yl diphosphate synthase (HDS), 5 unigenes for 1-deoxy-d-xylulose 5-phosphate reductoisomerase (DXR), 5 unigenes for 1-deoxy-d-xylulose-5-phosphate synthase (DXS), 4 unigenes for geranyl pyrophosphate synthase (GPPS), 1 unigene for accetyl-CoA acetyltransferase (ACAT), 1 unigenes for mevalonate kinase (MVK), 1 unigenes for phosphomevalonate kinase (MPVK), 1 unigenes for isopentenyl diphosphate isomerase (IPPI), 1 unigenes for 2-C-methyl-d-erythritol 4-phosphate cytidylyltransferase (MEPCT), and 1 unigene for 2-C-methyl-d-erythritol-2, 4-cyclodiphosphate synthase (MECPS). Most of the above unigenes were also identified in the “terpenoid backbone biosynthesis” pathway in Atractylodes lancea (Ahmed et al. 2016), a traditional Chinese medicine, and Cymbopogon flexuosus (lemongrass) (Meena et al. 2016). This indicates that the major component and the biosynthesis of volatile oil in different aromatic plants are common.

**Fig. 4:**
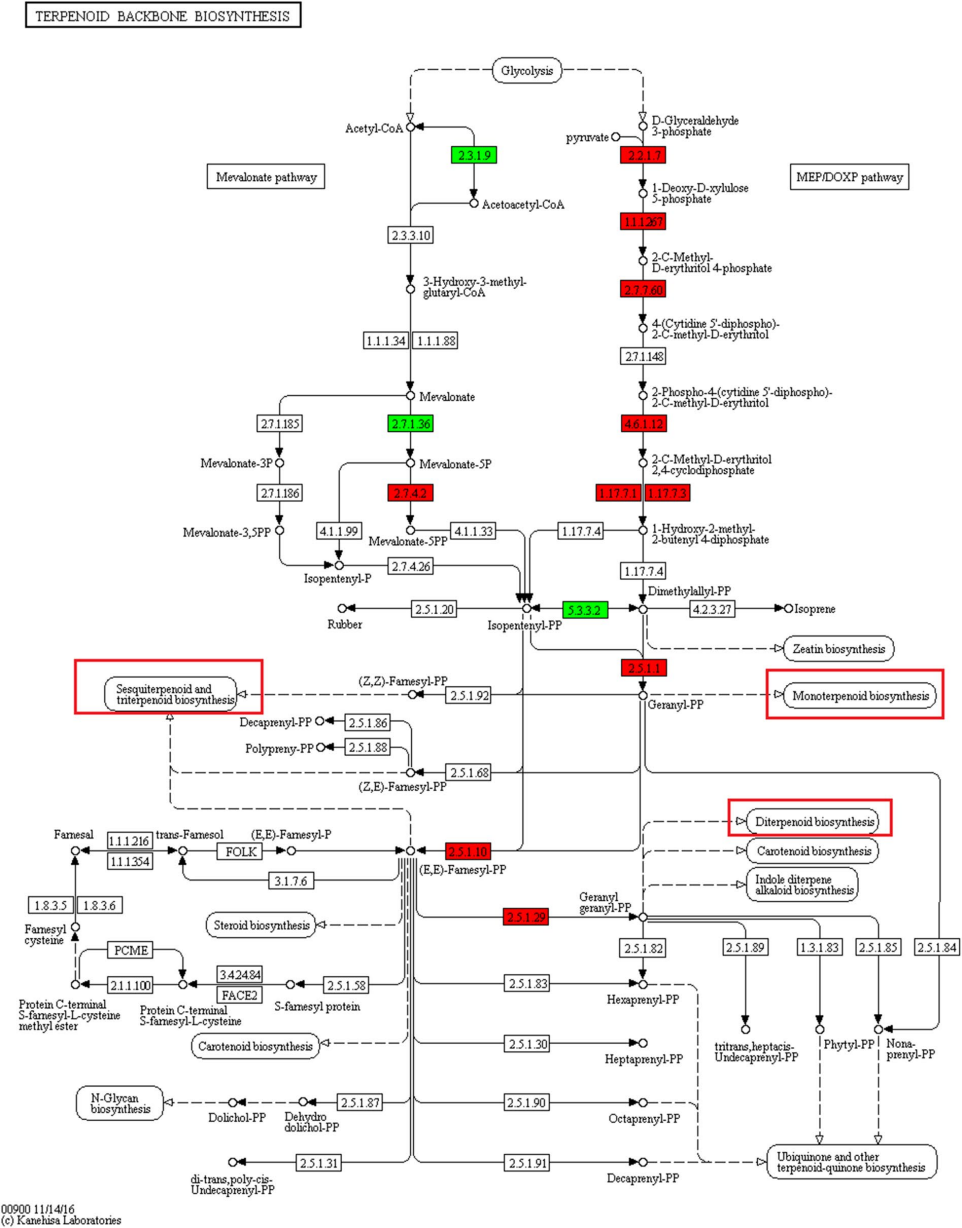
Differentially expressed genes (DEGs) involved in the “terpenoid backbone biosynthesis” pathway of ginger. The red columns indicate genes expressed at a significantly high level in fibrous roots. The green columns indicate genes expressed at a significantly high level in rhizomes. The red boxes indicate the biosynthesis of the major compounds found in ginger volatile oil. This color-coded map of DEGs corresponds to map00900 in the KEGG database (http://www.genome.jp/dbget-bin/www_bget?pathway:map00900)

The “terpenoid backbone biosynthesis” pathway includes two biosynthetic pathways, the mevalonate pathway and the non-mevalonate pathway (MEP/DOXP) pathway (KEGG). These two pathways co-exist in higher plants but operate in separate cellular compartments. The mevalonate pathway operates in the cytosol and gives rise to triterpenes, sterols, and most sesquiterpenes. The MEP/DOXP pathway operates in the plastids and mainly synthesizes essential oil monoterpenes and linalyl acetate, some sesquiterpenes, diterpenes, carotenoids, and phytol (http://www.genome.jp/dbget-bin/www_bget?pathway:map00900). Sesquiterpenoids and monoterpenoids are major biochemical constituents of ginger volatile oil (Jeena et al. 2013; Mashhadi et al. 2013). In this study, three unigenes (ACAT, EC:2.3.1.9, MVK, EC:2.7.1.36, and MPVK, EC:2.7.4.2) mapped to the mevalonate pathway, and two of them (ACAT and MVK) expressed to a greater degree in both matured and young ginger rhizomes than in fibrous roots (Fig. 4). In contrast, more unigenes were mapped to the MEP/DOXP pathway than to the mevalonate pathway, and the majority of these unigenes were expressed to a significantly greater degree (log_2_ (fold-change) > 1) in fibrous roots than in rhizomes, such as HDS (EC:1.17.7.1 and 1.17.7.3) and GPPS (EC:2.5.1.1, 2.5.1.10, and 2.5.1.29) (Fig. 4). This result suggests that the biological process responsible for the biosynthesis of volatile oil is different between ginger rhizomes and fibrous roots, and the fibrous roots play important roles in the biosynthesis of ginger volatile oil.

**Fig. 5:**
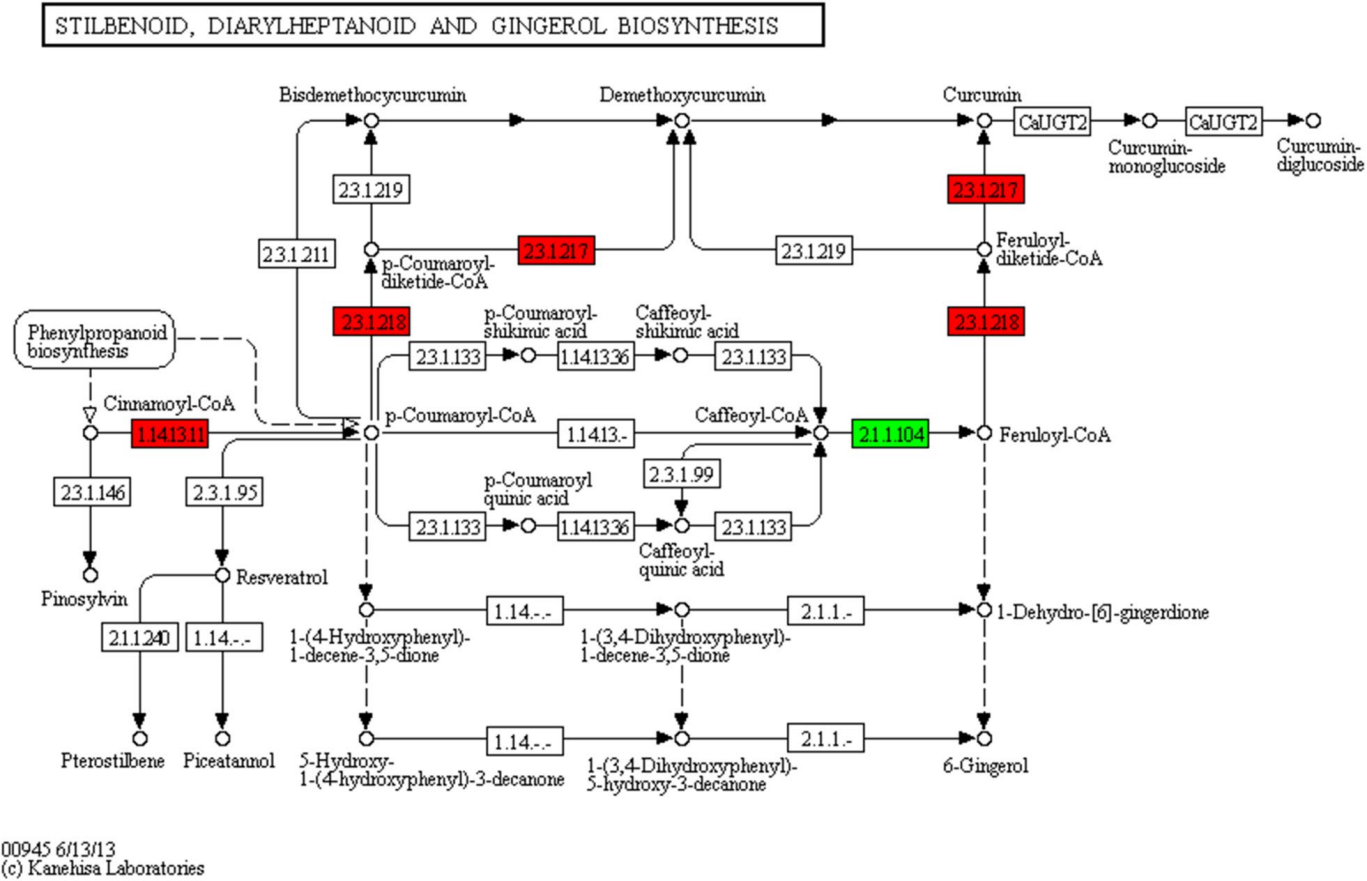
Differentially expressed genes (DEGs) involved in the “stilbenoid, diarylheptanoid and gingerol biosynthesis” pathway. The red columns indicate genes expressed at a significantly high level in fibrous roots; the green columns indicate genes expressed at a significantly high level in rhizomes. This color-coded map of DEGs corresponds to map00945 in the KEGG database (http://www.genome.jp/dbget-bin/www_bget?pathway:map00945)

In addition, a total of 23 unigenes “stilbenoid, diarylheptanoid and gingerol biosynthesis” pathways (Additional file 6: Table S4), including 12 unigenes for curcumin synthase (CURS), 5 unigenes for trans-cinnamate 4-monooxygenase (CYP73A), 5 unigenes for phenylpropanoylacetyl-CoA synthase (DCS), and 1 unigene for caffeoyl-CoA O-methyltransferase (CCOMT). The “stilbenoid, diarylheptanoid and gingerol biosynthesis” pathway is responsible for the biosynthesis of gingerols and diarylheptanoid. These compounds exhibit a host of biological and pharmacological activities, including anticancer, anti-oxidant, antimicrobial, anti-inflammatory and anti-allergic activities (Dugasani et al. 2010; Jiang et al. 2007; Mashhadi et al. 2013; Ramirez-Ahumada et al. 2006; Semwal et al. 2015). In this study, none of the 23 unigenes mapped to the “stilbenoid, diarylheptanoid and gingerol biosynthesis” pathway was directly related to the biosynthesis of gingerols (Fig. 5). This is consistent with the previous study by Ramirez-Ahumada et al. (2006), which reported that no gingerol synthase activity could be identified in extracts from ginger rhizomes. However, unigenes involved in the biosynthesis of curcuminoids were expressed at higher levels in fibrous roots than in rhizomes (Fig. 5). Curcuminoids are diarylheptanoid compounds found in turmeric, and represent an important category of biochemical components (Jiang et al. 2007). This indicates that diarylheptanoids are predominantly synthesized in fibrous roots.

In addition to these important bioactive compounds, ginger contains a large amount of flavonoids, which are important pharmacologically active metabolites that display antioxidant, anti-aging, and anti- cancer properties (Ghasemzadeh et al. 2010b; Knekt et al. 2002). In this study, 12 unigenes were mapped to “flavonoid biosynthesis” (ko00941) pathways (Additional file 6: Table S4). In contrast to the expression patterns of unigenes involved in the biosynthesis of volatile oil and diarylheptanoids, most unigenes related to the biosynthesis of flavonoids were expressed most highly in young ginger (Additional file 6: Table S4). The differential expression of genes related to the synthesis of biologically and pharmacologically active compounds in different ginger tissues suggests that, although these compounds accumulate in ginger rhizomes, they may be synthesized in other tissues. The synthesis, transport, and storage of these compounds require further investigation.

### Expression validation by qPCR

**Fig. 6:**
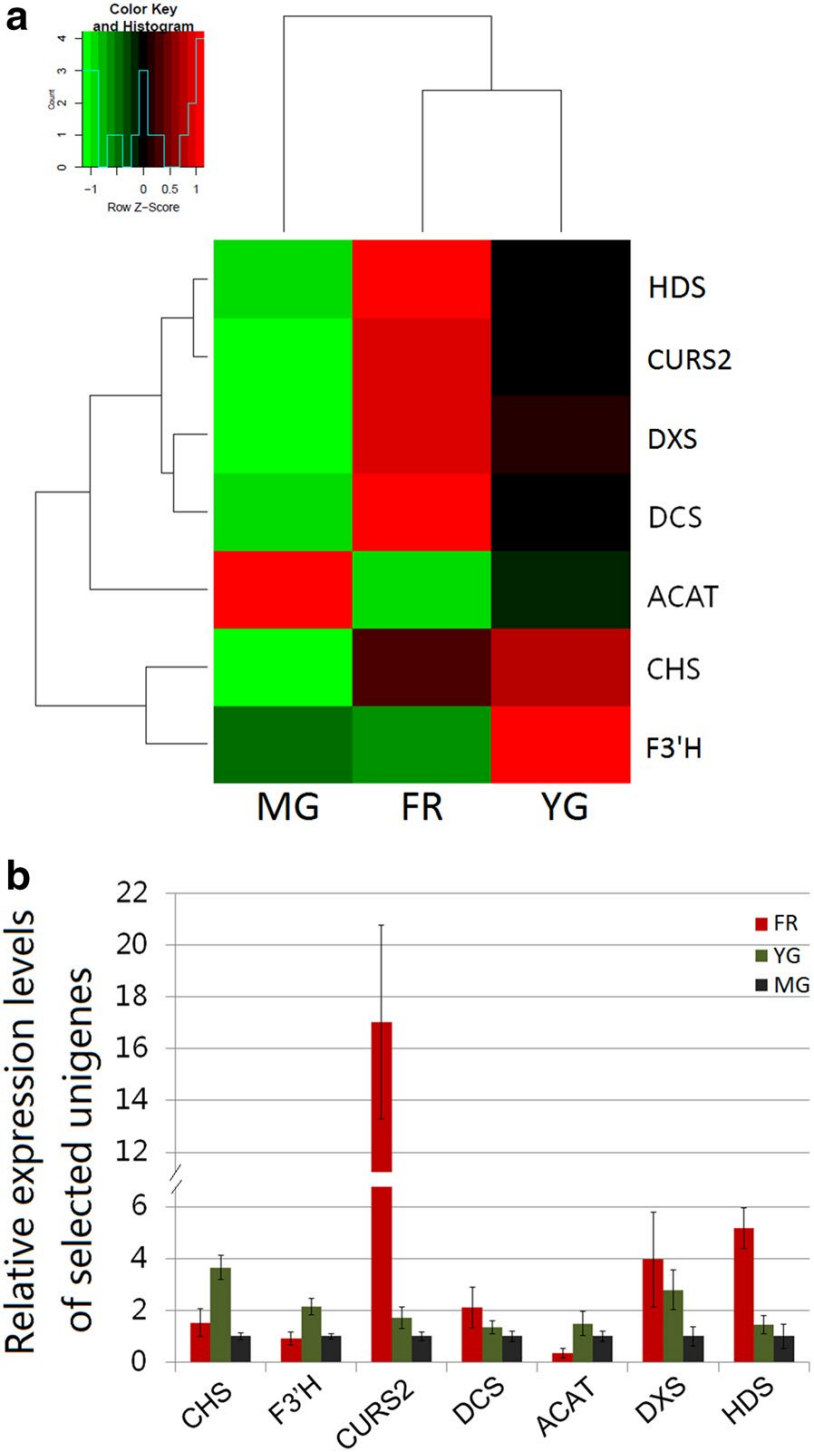
Heat map of the expression levels of seven ginger unigenes as determined by the fragments per kilobase of exon per million fragments mapped (FPKM) (**a**) and qRT-PCR analysis (**b**)

To evaluate the expression profiles of DEGs obtained by transcriptome sequencing, seven DEGs, including two DEGs (chalcone synthase, CHS, and flavonoid 3′,5′-hydroxylase, F3′H) in the “flavonoid biosynthesis” pathway (ko00941), two (curcumin synthase 2, CURS2, and phenylpropanoylacetyl-CoA synthase, DCS) in the “stilbenoid, diarylheptanoid and gingerol biosynthesis” pathway (ko00945), and three (acetyl-CoA acetyltransferase, ACAT, 1-deoxy-d-xylulose-5-phosphate synthase, DXS, and 4-hydroxy-3-methylbut-2-en-1-yl diphosphate synthase, HDS) in the “terpenoid backbone biosynthesis” pathway (ko00900), were selected for expression validation using qRT-PCR analysis (Additional file 3: Table S1). These three pathways are of the major pathways that are responsible for the biosynthesis of bioactive compounds in ginger such as flavonoid and volatile oil. The expression levels calculated by qRT-PCR were consistent with those from transcriptome sequencing data (Fig. 6). This indicated that the transcriptome sequencing is an effective way to investigate the differences on genes expressions and biological processes between different tissues.

## Conclusion

De novo transcriptome sequencing and assembly of three ginger tissues (MG, YG, and FR) generated a total of 361,876 unigenes, with an average length of 515.89 bp. Pairwise comparisons of unigene expression levels indicated that the unigenes’ expression pattern were similar between different parts of ginger rhizomes (YG versus MG), but different between ginger rhizomes and fibrous roots (YG versus FR, and MG versus FR). In addition, KEGG pathway enrichment for DEGs between different ginger tissues suggested that pathways responsible for the biosynthesis of volatile oil, diarylheptanoids, and substrates of these compounds, were predominantly represented. Moreover, most of the unigenes mapped in these pathways expressed significantly higher in FR than in YG or MG. Our result established that bioactive compounds in ginger may be predominantly synthesized in fibrous roots and then transported to rhizomes, where they accumulate. This study provides the first insight into the biosynthesis of bioactive compounds in ginger at a molecular level and provides valuable genome resources for future molecular studies on ginger.

## Additional files

**Additional file 1: Figure S1.** The length distribution of the assembled ginger transcriptome.

**Additional file 2: Figure S2.** A Venn diagram showing annotation of all of the assembled ginger unigenes that were found in different databases (A) and the unigenes that matched the 15 top species in the NR database (B).

**Additional file 3: Table S1.** Summary of unigene expression levels in ginger matured rhizome, young rhizome, and fibrous root libraries using the fragments per kilobase of exon per million fragments mapped (FPKM) method.

**Additional file 4: Table S2.** Significantly enriched Kyoto Encyclopedia of Genes and Genomes (KEGG) pathways of differentially expressed genes identified from pairwise comparisons between matured and young rhizome, young rhizome and fibrous root, matured rhizome and fibrous root libraries.

**Additional file 5: Table S3.** KEGG enrichment of DEGs between libraries of MG and YG.

**Additional file 6: Table S4.** Unigenes mapped to the pathways that responsible for the biosynthesis of major bioactive compounds in ginger.

## Abbreviations

ACAT: accetyl-CoA acetyltransferase
CCOMT: caffeoyl-CoA *O*-methyltransferase
CDS: coding sequence
CHS: chalcone synthase
CURS: curcumin synthase
CYP73A: trans-cinnamate 4-monooxygenase
DCS: phenylpropanoylacetyl-CoA synthase
DXPS: 1-deoxy-d-xylulose-5-phosphate synthase
DXR: 1-deoxy-d-xylulose 5-phosphate reductoisomerase
DXS: 1-deoxy-d-xylulose-5-phosphate synthase
F3′H: flavonoid 3′,5′-hydroxylase
FR: fibrous roots
GPPS: geranyl pyrophosphate synthase
HDS: 4-hydroxy-3-methylbut-2-en-1-yl diphosphate synthase
IPPI: isopentenyl diphosphate isomerase
MECPS: 2-C-methyl-d-erythritol-2, 4-cyclodiphosphate synthase
MEPCT: 2-C-methyl-d-erythritol 4-phosphate cytidylyltransferase
MG: matured ginger rhizome
MPVK: phosphomevalonate kinase
MVK: mevalonate kinase
ROS: reactive oxygen species
TCM: traditional Chinese Medicine
UDG: uracil-DNA glycosylase
YG: young ginger rhizome
