# Supplementary figures and images for: Transcriptome analysis reveals the genetic basis underlying the biosynthesis of volatile oil, gingerols, and diarylheptanoids in ginger (Zingiber officinale Rosc.)
Source: Bot Stud. 2017 Oct 23;58:41. doi: 10.1186/s40529-017-0195-5 (PMC5651534; doi:10.1186/s40529-017-0195-5)

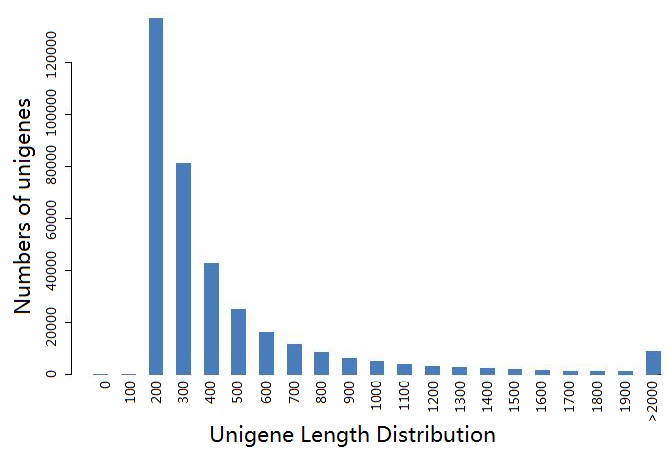

Supplement: Supplementary file 1 — Additional file 1: Figure S1. The length distribution of the assembled ginger transcriptome. [file 40529_2017_195_MOESM1_ESM.jpg]

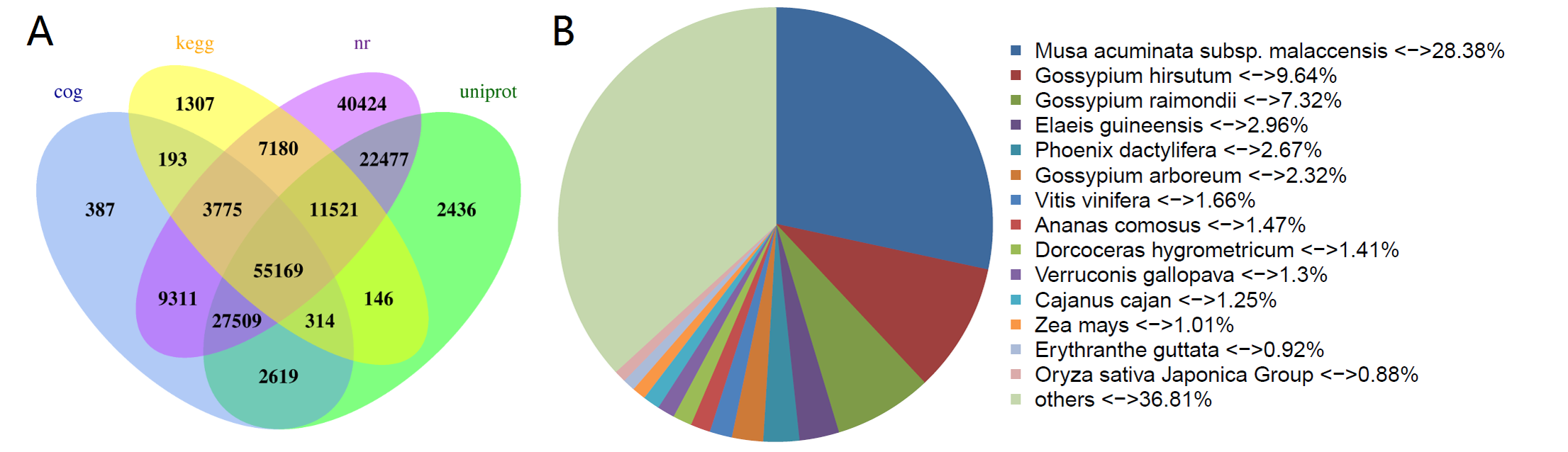

Supplement: Supplementary file 2 — Additional file 2: Figure S2. A Venn diagram showing annotation of all of the assembled ginger unigenes that were found in different databases (A) and the unigenes that matched the 15 top species in the NR database (B). [file 40529_2017_195_MOESM2_ESM.tif]
